# Recent advances in the functional explorations of nuclear microRNAs

**DOI:** 10.3389/fimmu.2023.1097491

**Published:** 2023-02-22

**Authors:** Xiaozhu Hu, Guoquan Yin, Yuan Zhang, Liangyu Zhu, Haoyu Huang, Kun Lv

**Affiliations:** ^1^Central Laboratory, The First Affiliated Hospital of Wannan Medical College, Wuhu, China; ^2^Key Laboratory of Non-Coding RNA Transformation Research of Anhui Higher Education Institutes (Wannan Medical College), Wuhu, China

**Keywords:** nuclear microRNA, post-transcriptional, gene regulation, nuclear function, NamiRNA–enhancer–gene activation network

## Abstract

Approximately 22 nucleotide-long non-coding small RNAs (ncRNAs) play crucial roles in physiological and pathological activities, including microRNAs (miRNAs). Long ncRNAs often stay in the cytoplasm, modulating post-transcriptional gene expression. Briefly, miRNA binds with the target mRNA and builds a miRNA-induced silencing complex to silence the transcripts or prevent their translation. Interestingly, data from recent animal and plant studies suggested that mature miRNAs are present in the nucleus, where they regulate transcriptionally whether genes are activated or silenced. This significantly broadens the functional range of miRNAs. Here, we reviewed and summarized studies on the functions of nuclear miRNAs to better understand the modulatory networks associated with nuclear miRNAs.

## 1 Introduction

Small non-coding RNAs (ncRNAs), which range in length from 20 to 22 nucleotides, including microRNAs (miRNAs), are among the most conserved ncRNAs. Several studies have confirmed that miRNAs are critical modulators of gene expression (1–3) (namely, proliferation, differentiation, apoptosis, and stress reactivity) and numerous diseases (4–7) (namely, neurological diseases, cardiovascular diseases, cancer, and aging). The nucleus and cytoplasm are involved in the multi-step process of miRNA synthesis. The most primitive miRNAs have long primary miRNAs (pri-miRNAs) with caps and polymeric A tails. A protein complex and microprocessor subsequently process these to form precursor miRNAs (pre-miRNAs). The pre-miRNAs are then released into the cytoplasm using Exportin5 (XPO5), where they are cleaved by RNaseIII type protein (Dicer) to produce double-stranded miRNAs. One of the two miRNA strands matures in the cytoplasm and forms the RNA-induced silencing complex (RISC), while the other degenerates instantly (8, 9). Since miRNAs only mature in the cytoplasm, it was previously determined that miRNA action was cytoplasm-specific. However, identifying miRNAs in the subcellular compartments of the nucleus reveals various novel miRNA-based functions in cellular homeostasis, which has been the subject of an increasing number of studies, some of which used data from small RNA deep sequencing (10). In this article, we described how nuclear miRNAs affected gene transcription and elaborated on the associated signal transduction in the nucleus.

## 2 Subcellular localization of miRNAs

Pre-miRNAs are released into the cytoplasm by a specific nuclear transporter (i.e., XPO5) in the classical miRNA processing pathway, where they mature. Therefore, scientists initially speculated that mature miRNAs were restricted to the cytoplasm. The modulatory role of miRNA in the nucleus has been verified by emerging evidence from recent reports (11). The majority of nuclear miRNAs articles that have been published are summarized in **Table 1**.

**Table 1:** The most of published literatures about nuclear miRNAs.

Nuclear miRNA	Species	Validated target promote(s)	Gene regulatory effect	Ref.
miR-709,miR-706, miR-690,	Mouse	MPRO, EL4,A20	Activation	(11)
miR-467a				
miR-21	Human	EFRP	Silencing	(12)
miR-206	Mouse	28S rRNA	Silencing	(13)
miR-122	Human	SQMB	Activation	(14)
miR-32, miR-148a, miR-29b,	Human	NA	NA	(15)
miR-148b, miR-1,miR-1285,				
miR-652, miR-29c,miR- 15b,				
miR-135b				
miR-342-3p, miR-345	Human	ELL2, HLF	Silencing	(16)
miR-3535,	Mouse	E2F3, HOXA3,XIST	Activation	(17)
miR-1291,miR-210-3p				
miR-20a	Human	E2F1-3	Activation	(18)
miR-709, miRNA-15a/16-1	Mouse	Dleu2	Silencing	(19)
miR-122	HCC cell	ERp72	Silencing	(20)
miR-373,miR-320,miR-423-5p	Human	Ccnb1	Activation	(21)
,miR-372,miR-373,miR-520c-				
3p				
miR-744,miR-1186,miR-466d-	Mouse	Ccnb1	Activation	(21)
3p,				
miR-665	Mouse	PTEN	Activation	(22)
miR-320	Mouse	SRF,CD36	Silencing	(23)
miR-320	Mouse	CD36	Silencing	(24)
miR-133a	Human	DNMT3B	Silencing	(25)
miR-30,miR-21	Human	Exp5	Silencing	(26)
miR-664,miR-351,miR-21,mi	Mouse	NA	NA	(27)
R-1,miR-206,miR-let-7a,miR-				
494,miR-125b-5p,miR-199a-3				
p				
miR-351,miR-494,miR-340,mi	Mouse	IGF2	Activation	(28)
R-206-3p,miR-206-5p, miR-30b-5p	Human	TFEB	Silencing	(29)
microRNA-466c	Human	VEGFR	Activation	(30)
miR-552-3p	Mouse	FXR, LXR	Activation	(31)

Major miRNAs with a reported function in the nucleus, including the species, their validated targets and promoters, biological function, and corresponding reference.

Meister et al. showed in 2004 using Hela cell that mature miR-21 can be found in the cytoplasm and the nucleus but that 70-nt pre-miR-21 is only found in the nucleus. This unique discovery of mature nuclear miRNA sparked the hypothesis that some mature nuclear miRNA can re-enter the nucleus (12). In a subsequent investigation, Politz et al. presented visual evidence of mature miRNA expression in the nucleus (13). They used nucleic acid probe based *in situ* hybridization analysis, in particular, to reveal that miR-206 aggregated in the nucleoli. However, a probe complementary to the precursor miR-206 showed no nucleolar signal. Moreover, they found that the granular component was the precise location of nuclear miRNA expression. In 2009, Papp et al. used femtosecond laser microscopy to investigate multiple single living cells exploiting the association between super quencher molecular beacon probes and human single-stranded cellular miR-122 targets. For the first time, they demonstrated that mature miR-122 penetrates the nucleus of human hepatocytes after being assembled in the cytoplasm (14). Massive advancements in high-throughput analyses over the past ten years have facilitated the discovery that mature nuclear miRNAs are widespread in most mammalian cells (15, 16). In 2019, Turunen et al. used a mouse endothelial cell line to perform nuclear-cytoplasmic fractionation and small RNA sequencing analysis. They identified 196 miRNAs (56% of all miRNAs), which were exclusively distinct between the nucleus and the cytoplasm. This suggested that miRNAs had a compartmentalized nature. Here, both compartments demonstrated a ≥2-fold enrichment of 105 miRNAs (specifically, nucleus: 46 and cytoplasm: 59). According to the M versus A plot analysis, the differential enrichment was apparent throughout a broad range of absolute expressions (i.e., counting). Nuclear miR-3535 (27.9-fold enrichment) and cytoplasmic miR-27a-5p (48-fold enrichment) were the top compartment-specific miRNAs. The content of miR-210-3p elevates in the nucleus after hypoxia (17). Given this extensive body of evidence, there is good reason to speculate that miRNAs have a functional role in the nucleus.

## 3 Functions of nuclear miRNAs

**Figure 1:**
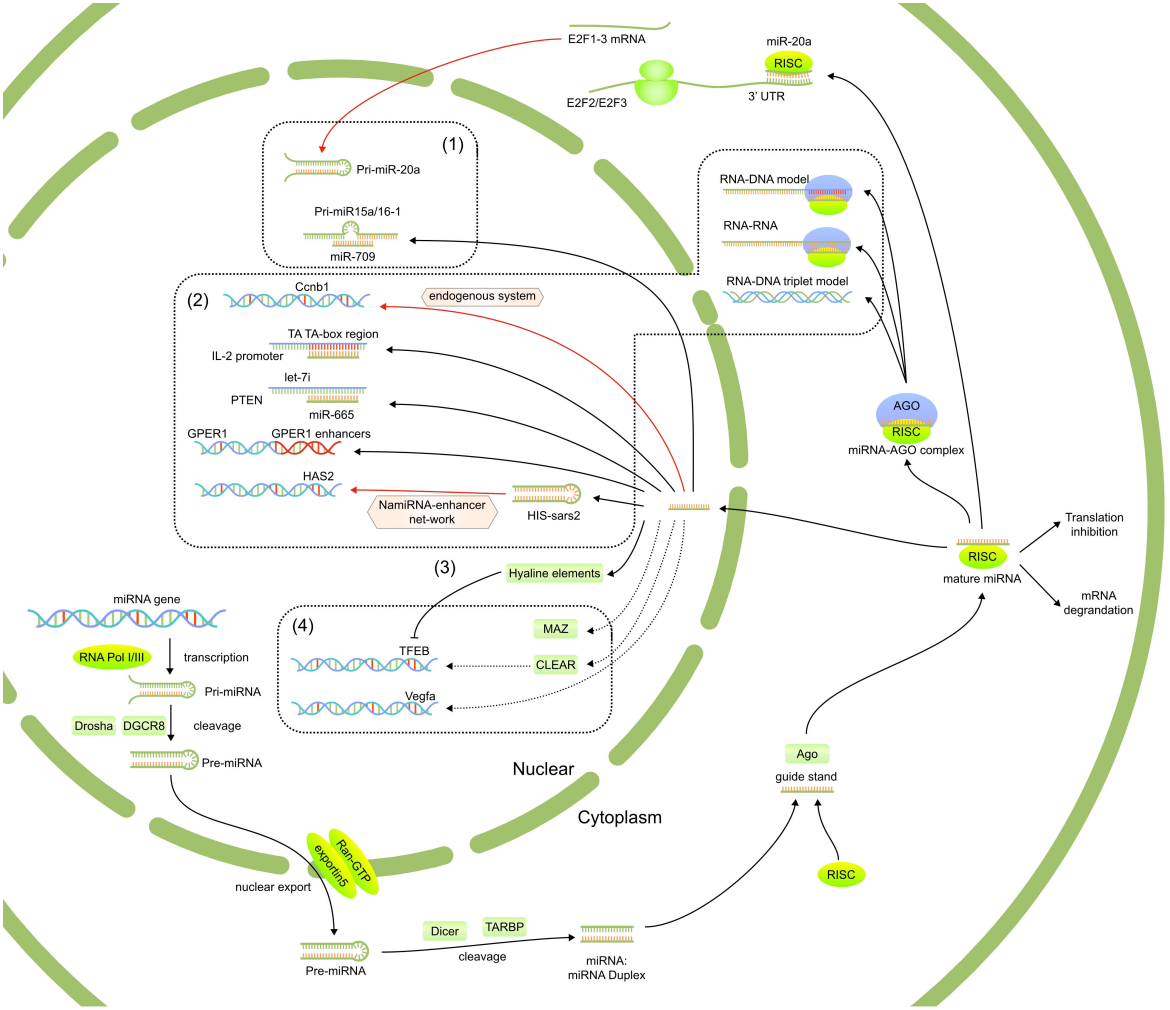
The reported functions of nuclear miRNAs, including 1)regulated the biogenesis and fuctions of other miRNAs, 2) targeted gene promoter or enhanced elements, 3) affect post-transcriptional regulation of mRNAs in the cytoplasm through nuclear translocation and 4) regulate potential signaling pathways.

**Figure 2:**
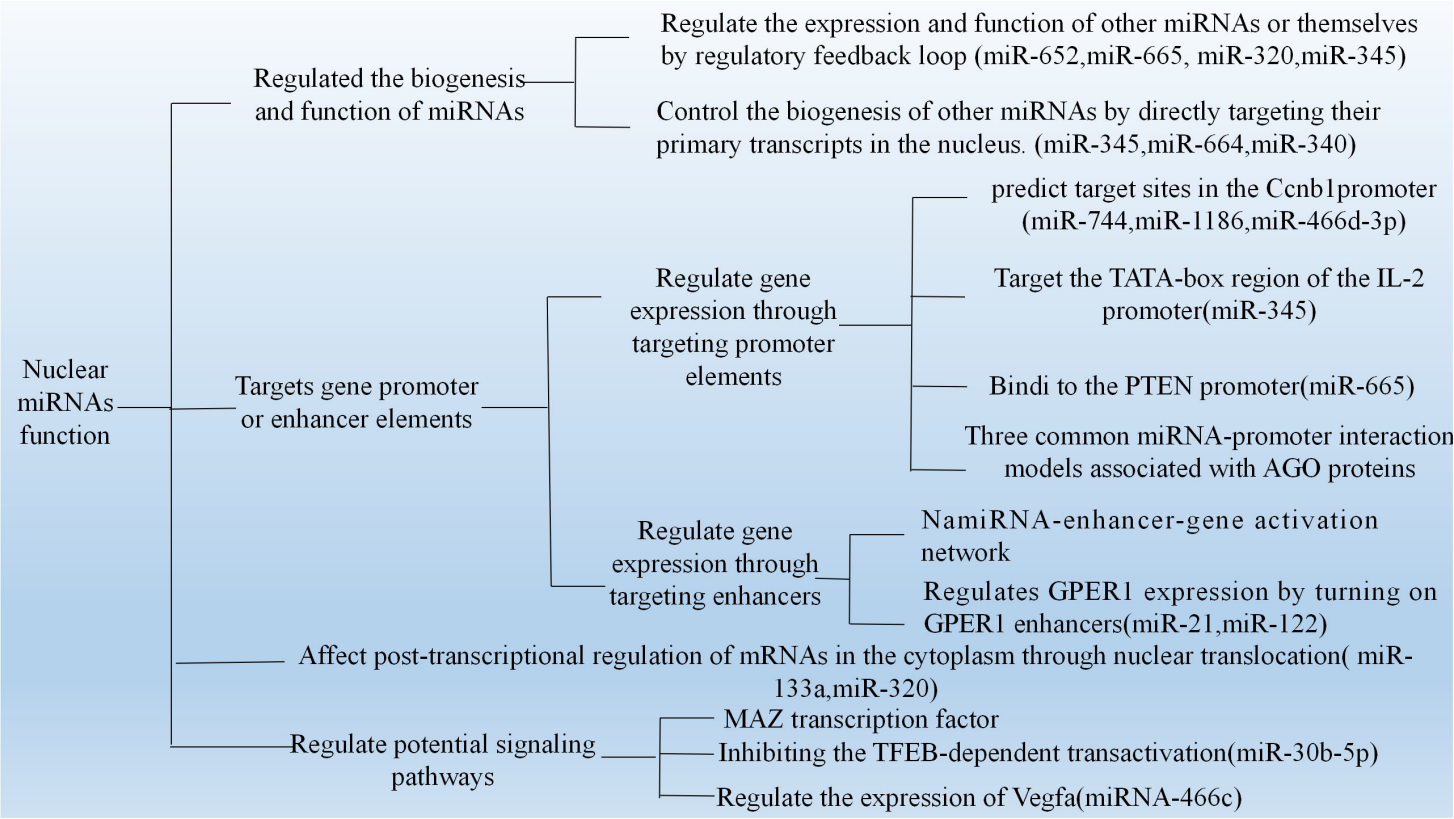
The mechanism of nuclear miRNAs which have been published. (1) The strong modulatory feedback loop between E2F1-3 and miR-20a. (2) Nuclear miRNAs target gene promoter or enhancer elements. (3) Participation of TFs for nuclear miRNA function. (4) Interact with the transcript to modulate Vegfa expression.

It is well known that miRNAs co-localize with argonaute (AGO) proteins in the nucleus, which is strongly suggestive of a role for miRNAs in modulating gene expression. In recent years, many nuclear miRNAs functions have been reported, including the direct regulation of miRNAs biogenesis and association with target gene promoters or enhancers. **Figures 1**, **2** present a summary of the reported functions of nuclear miRNAs.

### 3.1 Nuclear miRNAs directly regulate miRNA biogenesis and function

New evidence suggests that miRNAs indirectly regulate the expression and functions of both their own and other miRNAs. There is evidence of a potent modulatory feedback loop between E2F1-3 and miR-20a. Briefly, miR-20a regulates E2F2 and E2F3 translation *via* interacting with the 3′-untranslated region of each mRNA. Endogenous E2F1-3 transcript activates miR-20a transcription (18). Furthermore, several studies found that some miRNAs influence the fate of downstream miRNAs. In 2012, Zisoulis et al. (32) reported that the miRNA complex regulates ncRNAs, critical substrates in miRNA processing. They also demonstrated that let-7 miRNA primary mRNA interacts with the *Caenorhabditis elegans* AGO protein, ALG-1, at a specific 3′ end site to promote downstream processing events. This association creates a positive-feedback loop by using the mature let-7 miRNA through a conserved complementary site on its primary mRNA. Furthermore, AGO associates with the let-7 primary mRNAs in human cells, supporting the idea that miRNAs also target ncRNAs in addition to protein-coding mRNAs in different species. It also shows how AGO aids in the biogenesis of target mRNA. These reports suggest miRNA may play a modulatory role in its nuclear-based biogenesis.

In addition, Tang et al. found in 2012 that miRNA directly associates with primary mRNAs in the nucleus to regulate the production of other miRNAs (19). They demonstrated a negative correlation between miR-709, miR-15a, and miR-16-1 using miRNA microarray analysis in mice. Briefly, pre-miR-15a/16-1 processing into pre-miRNA is inhibited in the nucleus by directly binding miR-709. In 2018, Wang et al. (20), also confirmed that nuclear miR-122 inhibits miR-21 maturation by interacting with 19-nt UG in the basement region of pre-miR-21. These data collectively imply that miRNAs regulate the biogenesis of other miRNAs by binding to their primary mRNAs in the nucleus.

### 3.2 Nuclear miRNAs target gene promoter or enhancer elements

According to recent research, nuclear miRNAs can modulate gene expression by physically interacting with target promoter regions or by acting as an enhancer trigger. In 2012, Hang et al. evaluated several miRNAs in mice to estimate the target site within the Cyclin B1 (Ccnb1) promoter for inducing gene expression. They identified three distinct miRNAs (miR-744, miR-1186, and miR-466D-3p) that promote *Ccnb1* expression in murine cell lines, indicating an endogenous miRNA-based activation of Ccnb1 expression, which may be correlated with chromosomal instability (33). The same year, Huang and his colleagues (21) reviewed the literature on the role of nuclear miRNAs and looked at studies on how miRNAs affect transcriptional regulation. They discussed the movement of miRNA, which is processed in the cytoplasm, to the nucleus, where it might regulate gene expression. They discovered that miRNAs bind promotor regions in human and murine cells to initiate gene expression. They concluded that the promoter-targeting miRNAs and double-stranded RNAs (dsRNAs) used for gene modulation share a similar mechanism. In 2014, Zhang et al. showed a direct association between let-7i and the TATA-box of the IL-2 promoter (34). Given the great specificity of the miRNA and TATA-box motif interaction, it is feasible that miRNA-mediated transcriptional modulation is more precise than transcription factors (TFs) regulation.

In 2019, Fan et al. (22) reported miR-665 expression in the nucleus of cardiomyocytes. They demonstrated that high miR-665 expression exacerbated cardiac dysregulation brought on by transverse aortic constriction, whereas miR-665 had contrasting results. According to their findings, nuclear miR-665 substantially correlated with the phosphatase and tensin homolog (PTEN) promoter to accelerate heart failure by suppressing PTEN, suggesting potential treatment options for heart failure. In 2020, Zhan et al. reported that miR-320 regulates the transcription of several genes in the cytoplasm and the nucleus. In turn, this permits lipid aggregation in the cells of the heart and the liver (23).

Nuclear miRNAs also bind enhancers in addition to promoters. In 2017, Yu et al. discovered and defined a class of RNA-nuclear-activating miRNA (NamiRNA) with an activation function in the nucleus. They also proposed a novel theory of NamiRNA-enhancer-gene activation (NEGA) to understand better the interactions between NamiRNA and enhancers during target gene transcriptional regulation (35). According to Liang et al., the NEGA axis is crucial for cell identification throughout the transformation from normal to cancerous cells (36). They showed a strong association between miRNA and enhancers. MiRNA associates with the target enhancer to activate the gene, a process highly dependent on enhancer integrity.

Since late 2019, SARS-CoV-2 has caused disease with various clinical symptoms. Unfortunately, there have only been a few publications on the mechanism underlying the interaction of SARS-CoV-2 with the host and the emergence of subsequent symptoms. Li et al. (37) found five short sequences (24–27 nt) that include the human identical sequences (HIS) within the SARS CoV-2 genome. It revealed that the HIS-SARS2-mediated host gene activation uses inflammation *via* the NEGA network. It is generally known that HIS-containing genomic fragments form hairpin structures in silicon wafers, similar to the miRNA precursors. Therefore, HIS may activate host enhancers by direct interaction with the genome or by enhancing adjoining or distant genes like cytokine genes and hyaluronic acid synthase 2. These data give fresh perspectives on the development of COVID-19 therapies and indicate potential pathogenic signal transduction pathways behind the progression of the disease. In cancer, tumor suppressor gene (TSG) dysregulation is prevalent. Ying et al. (38) reported that miR-339 increases GPER1 levels in breast cancer cells by activating its enhancers, a process severely impaired by CRISPR/Cas9 deletion. This shows that the TSG reactivation by enhancer switching holds significant promise as an alternative approach to treating breast cancer in the clinic.

Unfortunately, there is no consensus on the signaling pathways used for miRNA-mediated up- or down-regulation of genes *via* enhancers. AGO proteins have been proposed in three miRNA-promoter association models thus far. According to the RNA-DNA model, the miRNA-AGO complex physically associates with a DNA strand that has openly configured TATA box modifications or TF binding sites. Once several TFs and/or epigenetic modifiers have bound to the promoter region, RNA polymerase II can be recruited and/or modified on an epigenetic level. Non-coding transcripts with promoter regions are a component of the RNA-RNA model. Both sense and antisense mRNAs interact with miRNA-AGO complexes to recruit TFs and/or histone modifiers. In the RNA-DNA triplet model, miRNA and DNA combine to form a triple helix, which modifies the shape of the chromatin and affects the type of TFs that can bind to the DNA. For example, two studies published in 2019 revealed that the nuclear miR-133a/AGO and miR-320/AGO complexes strongly inhibit transcriptional activity (24, 25).

### 3.3 Participation of TFs for nuclear miRNA function

Additionally, nuclear miRNAs use TFs to mediate their effects. A major constituent of the RISC complex, the AGO protein, is strongly involved in the RNA interference process (39) and can directly form complexes with miRNAs or small interfering RNAs (siRNAs) to modify gene expression. Human cells have four distinct AGO proteins (AGO1-4) (40). Although the roles of AGO1, AGO3, and AGO4 have yet to be fully established, it is known that they all bind RNA. AGO1 and AGO3 modulate gene expression without AGO2 (41). However, AGO2 is the only AGO protein among the four AGO proteins that can specifically cleave RNA upon forming a complex with a miRNA. In human cells, AGO2 is reported to regulate gene transcription and alternative splicing (42).

GW and P bodies have been shown to contain the TNRC6 protein family, which was the first antigen recognition factor identified in the autoimmune serum of patients with motor and sensory neuropathy (43). The AGO protein forms a complex with the TNRC6 protein family through strong associations (44). A TNRC6 mutation or deficiency severely inhibits miRNA-mediated gene silencing in mammalian cells (45), and recent research has established that the TNRC6 protein family contributes significantly to the miRNA pathway (46).

Moreover, according to Yi et al., XPO5 is required for both the nuclear translocations of human pre-miRNAs and the function of miRNA. *In vitro*, XPO5 only forms strong associations with pre-miRNAs when exposed to Ran-GTP cofactors. The nuclear transport of short hairpin RNA, a synthetic pre-miRNA analog for the expression of siRNAs, is significantly dependent on XPO5 (26). Moreover, Zhang et al. demonstrated that *in vivio* binding of pri-miRNAs to the pri-miRNA processor, DCL1 is mediated by PRL1, a nuclear miRNA interacting protein. PRL1 reduces pri-miRNA contents, suggesting that PRL1 likely stabilizes pri-miRNAs and functions as a cofactor for DCL1 activity enhancement without affecting pri-miRNA transcription (47). Meanwhile, Karlsson et al. discovered the *Arabidopsis thaliana* K homology domain protein regulator of CBF gene expression 3 (RCF3) as a nuclear miRNA production-specific plant tissue cofactor. They found lower miRNA and miRNA-target levels in the vertex-enriched samples of RCF3 mutants compared to other tissues (48). Another report suggested that DGCR8 directly and stably associates with pri-miRNA using its tandem dsRNA binding domains. This further emphasizes the Drosha-DGCR8 complex functions in pri-miRNA processing (49).

## 4 Functions of miRNAs in the nucleolus

According to recent studies, pre- and mature miRNAs were found in significant amounts (27). Determining the potential roles of the nucleolar miRNAs has thus given rise to multiple hypotheses.

In 2014, Reyes-Gutierrez et al. used bioinformatic analysis to estimate canonically structured and thermodynamically stable associations between the IGF2 transcript and all five nucleolus-based miRNAs. Based on this, the nucleolus is a primary location for targeted mRNA-miRNA associations prior to cytoplasm transport (28). Before miRNAs are redistributed to the cytoplasm under genotoxic stress to initiate assembling the RISC complex as part of the genomic defense system, they may be stored in the nucleoli.

In 2016, Atwood et al. found that the smallest RISC (AGO2 and miRNA) may impact the ribosomal RNA (rRNA) in cells (50). AGO2-rRNA association is highly dependent on miRNA and sustained PoII activity, as shown by the minimal RISC to the sensitivity of 45s rRNA interaction to both Dicer suppression and actinomycin D exposure. It is possible to hypothesize the particular properties of mature ribosomes. Additionally, it has been shown that pri- and pre-miRNA potential undergo nucleolar A-to-I editing because the nucleoli contain many RNA editing enzymes, namely, adenosine deaminase. This alteration suppresses miRNA maturation, which minimizes the cellular availability of mature miRNAs (51).

In conclusion, the model mentioned above-associated miRNA with nucleoli, indicating a strong necessity for the miRNA’s nucleolar location for the cytoplasmic post-transcriptional regulation of target transcripts.

## 5 Potential signaling pathways associated with the nuclear miRNA regulation

It is still unknown how nuclear miRNAs-regulate gene expression in this manner. Recent research, however, has supported numerous potential processes thought to be involved in this process.

AGO2-related genes were significantly enriched with MAZ docking locations and neural function, according to a 2017 study by Goldie et al. The AGO1 mRNA, in contrast, associates with SC35 spliceosomes and is connected to the general metabolic processes (52). This suggests that the MAZ TF is associated with nuclear miRNA and affects the control of neuronal development through the AGO2-related miRISCs. The MAZ TF may be crucial to organize higher-level correlations between transcriptional and post-transcriptional controls in primate neurons.

In 2021, Guo et al. revealed 2021 that the TFEB-induced transactivation is greatly enhanced by miR-30b-5p knockdown using CRISPR/Cas9, thereby upregulating genes involved in autophagy and lysosomal biogenesis (29). They discovered that the miR-30b-5p interacts with the nuclear CLEAR elements to modify lysosomal biogenesis and autophagy, presumably suppressing TFEB-based transactivation.

In the mouse endothelial cell line C166, Laitinen et al. discovered in 2022 that the nuclear microRNA miR-466c regulates the production of vascular endothelial growth factor a (VEGFA) when the environment is hypoxic (30). Additionally, the miR-466c deletion in the CRISPR-Cas9 genome greatly hinders the hypoxia-induced increase in VEGFA content. They also discovered long non-coding RNAs linked to the murine VEGFA promoter and hypothesized that miR-466c physically interacts with the transcript to modify VEGFA expression.

However, because nuclear miRNA is a novel gene modulator, more research is necessary to understand the networks in miRNA-mediated control of target gene expression.

## 6 The mechanism of nuclear miRNAs

The function and underlying mechanism of nuclear miRNAs are currently being studied. Previous research showed that the human miR-29b is largely found in the nucleus compared to other animal miRNAs. The unique hexanucleotide terminal motif of miR-29b functions as a transferable nuclear localization element that regulates either its own or linked siRNAs nuclear enrichment.

It also explains the highly redundant common miRNAs 5′ sequences, which may play independent roles in response to cis-acting modulatory motifs (53). The IPO8 was also found to have a significant role in transporting mature miRNA from the cytoplasm to the nucleus. Without changing the total number of miRNAs present in the cell, IPO8 knockdown, in particular, significantly reduces the nuclear translocation of certain miRNAs that are generally enriched in the nucleus. The IPO8 and AGO2 complex interaction is also necessary for the nuclear translocation of mature miRNAs mediated by IPO8. AGO2 nuclear transfer is likewise reduced by IPO8 knockdown without changing the overall levels of AGO2 cells. The effectiveness of IPO8-mediated miRNA nuclear transport was significantly reduced when the combination of miRNAs and AGO2 was dissociated (54). A single molecule fluorescence-based evaluation of the subcellular translocation, integrity, and activity of miRNAs was developed by Sethuramasundaram et al. They discovered that where the selection, melting, and nuclear retention of the miRNA chain is dependent on AGO identity, the stability and activity of the miRNA are strongly influenced by the amount of AGO protein. These findings suggest that miRNA degradation competes with AGO loading and target association to modify the subcellular miRNA content for monitoring gene silencing (55).

Furthermore, Santovito et al. showed that miR-126-5p interacts with Mex3a on the surface of autophagic vesicles to create a miR-126-5p/AGO2 complex, enabling it to reach the nucleus. Meanwhile, it was discovered that Mex3a connects with the central U- and G-type regions of miR-126-5p with nanomolar affinity utilizing mutational and biophysical analyses. The miR-126-5p/AGO2 complex dissociates upon entry into the nucleus, and miR-126-5p then binds with caspase-3 in the same manner as its seed sequence. This limits the ability of caspase to regulate cell death and prevents caspase dimerization (56). Notably, the mechanism driving the nuclear enrichment of miRNAs is tightly linked to several widely used signaling networks. Normally, miR-133a translocates from the canonical Wnt pathway to the cardiomyocyte nucleus. The nuclear miR-133a/AGO2 complex interacts with the DNA methyltransferase 3B (DNMT3B) promoter’s complementary miR-133a target site, causing DNMT3B to repress the transcription of itself (25).

Separate nuclear and cytoplasmic compartments are used for the production of miRNAs. As reported earlier, miRNA maturation depends on the nuclear transfer of precursors. Pre-miRNAs frequently use XPO-5 as a carrier. the family of importin-β/karyopherin includes XPO-558 (57). The mechanisms behind the pre-miRNA specificity of XPO-5 have already been elucidated by structure-based research (58). Similar to other importin-β/karyopherin families of proteins, XPO-5 is composed of a Huntingtin-elongation-A subunit-TOR repeat sequence consisting of two antiparallel α-sheets linked by a turn and appears as a spiral structure overall. The importin-β/karyopherin family of proteins can adapt to various vectors due to the inherent flexibility of this configuration (59). XPO-5 recognizes pre-miRNAs with similar structural profiles as a nuclear transfer signal (60). Therefore, the biosynthesis of miRNAs includes nuclear transportation as an integral part. Pre-miRNAs require a particular and essential transport receptor, and XPO-5 is present in most investigated organisms (61). However, emerging evidence revealed that embargoes (EMB; an immediate relative of XPO-1), another export receptor for leucine-rich NES proteins, influence the nuclear-cytoplasmic delivery of pri-miRNAs in the nematode *Caenorhabditis elegans*, which lacks an immediate relative of XPO-5 (62),. Although the role of EMB in this process is unknown, growing evidence suggests that the different organisms may use various miRNA-mediated nuclear translocation pathways. More recently, it was discovered that decreased RANBP1 significantly reduces the expression of hsa-miR-18a, hsa-miR-183, and hsa-miR-106 (miRNAs) in colorectal cancer by preventing the nuclear transfers of the corresponding pre-miRNAs. This facilitates the pre-miRNAs to accumulate in the nucleus, which lowers the amount of mature miRNA present. Evidence suggests that RANBP1 modulates pre-miRNA nuclear transportation to affect colorectal cancer cell proliferation, invasion, and apoptosis (63). Fungal virulence in plants depends on the secretory protein VdSSR1 (secreted silencing blocker 1), which is produced from Flavobacterium flavum. Zhu et al. demonstrated that VdSSR1 chelates the ALY family of proteins, potent modulators of the TREX complex, to modulate the nuclear transfer of the AGO1-miRNA complex. This increases fungal toxicity in plants by lowering the cytoplasmic AGO1 protein and sRNA contents (64).

According to a previous study, AGO proteins interacted with a GW protein family member known as TNRC6A-C in mammals, which coordinates downstream gene-silencing processes (65). The cytoplasmic functions of TNRC6 and AGO proteins are rather well known. The nucleus contains both protein families as well. Contrary, the epigenetic modifiers were recruited by RISC and guided by miRNAs to particular loci in the genome (66). These findings improved the role that proteins play in nuclear miRNA functions.

## 7 The emerging field of nuclear miRNAs

Scientists are gradually learning new roles and functions of these small RNAs due to an extensive investigation into the function of nuclear miRNAs in recent years. According to Indrabahadur et al., the nuclear miR-let-7d joins with ncRNA to create the MiCEE complex, which causes the epigenetic silencing of bidirectional gene expression in tissues with a large genome. It is important as a key regulator of bidirectional gene transcription is thus confirmed by this (67). Furthermore, extracted chromatin RNA and large parallel sequencing revealed that let-7i miRNA substantially inhibits norepinephrine transporter *via* association with methyl-CpG-binding protein 2 (68).

Ohno et al. discovered that the nuclear miRNAs separate stalled Pol II from the DDX21-CDK9 complex in 2022. The aforementioned researcher’s ZMYND10 activation caused by miR-34a as a system of RNA activation (RNAa) assessment to find a significant regulator of RNAa (31). Numerous investigations have also shown that nuclear miRNA regulates disorders related to glycolipid metabolism. By altering LXRα and FXR transcriptional activities in the nucleus, miR-552-3p worsens hepatic glycolipid metabolic disorders. This offers proof that miR-552-3p is an effective therapeutic target against metabolic diseases (69).

## 8 Conclusion

Although the accepted thinking was that miRNAs govern mRNA stability and translation in the cytoplasm, subsequent studies have shown that they also have a regulatory role in the nucleus for transcription that involves enhancers and promoters.

An independent effect approach is the biggest drawback of the current study on nuclear miRNAs. It is essential to develop a more efficient experimental system to examine the importance and purpose of nuclear miRNAs. Additionally, the location, movement, and purpose of nuclear miRNAs could be clearer. Furthermore, it is still unclear which possible signaling pathway nuclear miRNA regulates. Therefore, additional study into these areas will undoubtedly result in a breakthrough, opening up new possibilities for creating nuclear miRNA-based therapies for treating human diseases.

## Author contributions

XH (first author): performed the data analyses and wrote the manuscript; GY: contributed to the conception of the study; YZ: data curation; LZ: contributed significantly to analysis and manuscript preparation; HH: helped perform the analysis with constructive discussions. All authors contributed to the article and approved the submitted version.

## Glossary

**Table:**

ADAR	adenosine deaminase
AGO	Argonaute
BMDM	bone marrow-derived macrophage
Ccnb1	Cyclin B1
Dnmt3b	DNA methyltransferase 3B
dsRBDs	dsRNA binding domains
dsRNAs	double-stranded RNAs
HAS2	hyaluronic acid synthase 2
HEAT	Huntingtin-elongation-A subunit-TOR
HIS	Human Identical Sequences
HSV-1	Herpes simplex virus 1
KH	K homology
lncRNAs	long non-coding RNAs
MA	M versus A
MeCP2	methyl-CpG-binding protein 2
miRISC	miRNA-induced silencing complex
miRNAs	microRNAs
NamiRNA	nuclear activating miRNA
ncRNAs	non-coding RNAs
pre-miRNAs	precursor miRNAs
pri-miRNAs	primary miRNAs
PTEN	phosphatase and tensin homolog
RCF3	REGULATOR OF CBF GENE EXPRESSION 3
RISC	RNA-induced silencing complex
RNAa	RNA activation
rRNA	ribosomal RNA
SQMB	superquencher molecular beacon
TAC	transverse aortic constriction
TFs	transcription factors
TSGs	tumor suppressor genes
UTR	untranslated region
Vegfa	vascular endothelial growth factor a
XPO5	Exportin5
